# Dosimetric effect of body contour changes for prostate and head and neck volumetric modulated arc therapy plans

**DOI:** 10.1002/acm2.12571

**Published:** 2019-03-29

**Authors:** Lingyue Sun, Charles Kirkby, Wendy Smith

**Affiliations:** ^1^ Department of Physics and Astronomy University of Calgary Calgary AB T2N 1N4 Canada; ^2^ Department of Medical Physics Tom Baker Cancer Centre Calgary AB T2N 4N2 Canada; ^3^ Department of Oncology University of Calgary Calgary AB T2N 1N4 Canada; ^4^ Department of Medical Physics Jack Ady Cancer Centre Lethbridge AB T1J 1W5 Canada

**Keywords:** body contour change, head and neck, prostate, VMAT plans

## Abstract

Body contour changes are commonly seen in prostate and head and neck (H&N) patients undergoing volumetric modulated arc therapy (VMAT) treatments, which may cause a discrepancy between the planned dose and the delivered dose. Dosimetrists, radiation oncologists or medical physicists sometimes are required to visually assess the dosimetric impact of body contour changes and make a judgment call on whether further re‐assessment of the plan is needed. However, an intuitive judgment cannot always be made in a timely manner due to the complexity of VMAT plans as well as the complicated forms of body contour changes. This study evaluated the dosimetric effect of body contour changes for prostate and H&N patients to help with clinical decision‐making. By analyzing the one‐dimensional spatial dose profiles from the original body and the body with different body contour deformations, rules of thumb for dose percentage change and isodose line shift due to body contour changes were ascertained. Moreover, based on dose distribution comparison using three‐dimensional gamma analysis, the response of the clinical prostate and H&N VMAT plans to body contour changes was assessed. Within center specific dose deviation tolerances, prostate patients who had less than 2 cm single side body contour change or less than 1 cm uniform body contour change were unlikely to need plan re‐assessment; H&N VMAT plans with less than 1 cm uniform body contour change or less than 1 cm shoulder superior–inferior positional change were also unlikely to trigger further evaluation. Dose percentage change and isodose line shift were considered independently from the problem of volume changes in this study, but clinically, both aspects must be considered.

## 1 INTRODUCTION

The general goal of radiation therapy is to deliver a specific conformal dose of ionizing radiation to a target volume(s) while minimizing the dose to the surrounding normal tissues and organs‐at‐risk (OARs). Volumetric modulated arc therapy (VMAT) makes this possible by modulating beam delivery to achieve steep dose gradients between the target volume and the OARs. At the same time, the conformal dose distribution and the steep dose gradient may make VMAT sensitive to anatomical variations or setup uncertainties and raise the concern of overdosing the OARs or partially missing the target volume.^1^, ^2^

In a conventional planning process, the patient's treatment plan is created based on the anatomy present in the planning computed tomography image set (p‐CT), which is typically taken 1 to 2 weeks before the start of radiation therapy. However, during the course of radiation therapy, the patient's anatomy may change in ways that cannot be corrected by image guided radiation therapy (IGRT). Examples for prostate patients include weight change, buttock flex, and abdominal position change.^3^, ^4^ Weight change, tumor shrinkage, and shoulder position variations are commonly seen in head and neck (H&N) patients.^5^, ^6^, ^7^, ^8^, ^9^ As a result, the patient's body contour on the treatment day can deviate from the p‐CT and this can be clearly visualized on the volumetric images (e.g., cone‐beam CTs) taken on the treatment day.

Essentially, body contour changes can cause changes in beam path length, entry angle, and degree of phantom scatter in a given field. When combined with the high conformality and steep dose gradients generated with VMAT, body contour changes can potentially lead to significant differences between planned and delivered dose. In most cancer centers, it is common for changes in body contour to trigger re‐assessment of the plan.^3^, ^10^, ^11^, ^12^ Dosimetrists, radiation oncologists or medical physicists need to make a judgment call on further examining the dosimetric impact of body contour changes based on the cone‐beam CT (CBCT) taken before treatment as well as the decision to reposition and treat the patient.

In the era of 3D conformal radiation therapy, the effect of body contour changes was commonly estimated by the tissue phantom ratio (TPR) for isocentric setups. For intensity modulated radiation therapy (IMRT), the effect could be approximated by the weighted TPRs for all the fields, which could be done on the fly. However, for VMAT plans, where the dose rate, gantry speed, and multileaf collimators' movements are changing during the 360° delivery around the patient, it is not intuitive to assess the dosimetric impact of body contour changes. One can perform full dose calculations on the CBCT image sets. This time consuming method can be problematic because of the lack of an accurate electron density conversion curve for CBCT systems and problems associated with image registration between the CBCT and p‐CT. On the other hand, the number of patients who can be re‐assessed and re‐planned is constrained by the limitation of resources in a clinic. Thus, it is important to have some efficient ways to evaluate the impact of body contour changes on the spatial dose distribution as well as knowing how sensitive the plans are to body contour changes.

In this study, we provide rules of thumb for dose percentage change and isodose line shift due to body contour changes for prostate and H&N VMAT plans. Our analysis is based on one‐dimensional dose profile comparison and 3D gamma analysis.

## 2 MATERIALS AND METHODS

### 2.A Patient population

Twelve early‐to‐intermediate stage prostate cancer patients (six prescribed with 78 Gy in 39 fractions and six prescribed with 60 Gy in 20 fractions) and ten loco‐regionally advanced oropharyngeal cancer patients [70 Gy in 33 fractions to the primary gross tumor and high risk nodal regions, namely, high risk clinical target volume (CTV_H) and 59.4 Gy in 33 fractions to low‐risk nodal region, namely, low risk clinical target volume (CTV_L)] were randomly selected. All of them were treated with VMAT and retrospectively analyzed.

### 2.B VMAT treatment planning

For prostate plans, the CTV was defined as the prostate with or without proximal seminal vesicles; the planning target volume (PTV) was a 10 mm expansion of the CTV, 7 mm posteriorly. For the oropharyngeal plans, the high dose PTV and low dose PTV were created by a 3 mm uniform expansion of the corresponding CTVs but the PTVs were cropped 3 mm from skin.

All the clinical plans were made by the dosimetrists in the treatment planning system using the progressive resolution optimizer (Eclipse version 11.0, Varian Medical System, Palo Alto, CA) as per PROFIT trial protocol (60 Gy/20fractions),^13^ departmental prostate protocol (78 Gy/39fractions),^14^ and departmental H&N protocol^14^. Each clinical plan had two to three full arcs with the energy of 6 MV and the anisotropic analytical algorithm with a dose grid resolution of 2.5 mm was used for dose calculation.

### 2.C Body contour deformation

To create CT image sets with deformed body contours and to assess the theoretical impact of body contour changes, body contours in the p‐CT were deformed using the margin tool in Eclipse Contouring for body contour shrinkage and expansion. For body contour expansion, the air gap between the original body and the new contour was assigned HU = 0 for simplicity. Although in reality, this part of the tissue may be mostly adipose tissue (mass density ~ 0.9 g/cm^3^) with typical HU in the range of −190 to −30,^15^ it is unlikely to greatly affect the dose deposition. In Eclipse, dose is calculated within the body contour structure and any material outside of the body contour is treated as vacuum (with the exception of designated support structures).

For prostate patients, the body contours were changed by ± 1 cm and ± 2 cm in four different ways: (a) in anterior direction only to simulate abdomen position change; (b) in posterior direction only to simulate the effect of buttock position change; (c) in left direction only to simulate lateral position change with the assumption that the patient was symmetrical in left and right direction; (d) in anterior, left, and right directions uniformly to simulate overall weight change.

**Figure 1:**
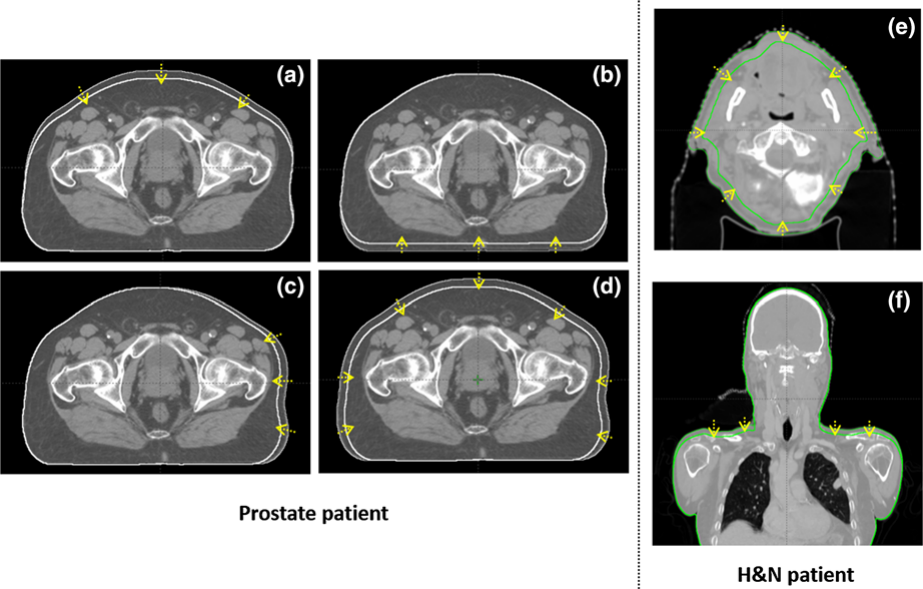
Examples of body contour changes for prostate and H&N patients shown in transverse view or coronal view. For prostate patients, the thin white contour was the original body contour and the bold white was the deformed body contour. The arrows were showing the direction of body contour changes. (a): anteriorly shrunk 1 cm; (b): posteriorly shrunk 1 cm; (c): left side shrunk 1 cm; (d): uniformly (anterior, left, and right) shrunk 1 cm. For H&N patients, the thin green contour was the original body contour and the bold green was the deformed body contour. (e): uniformly shrunk 1 cm in facial area; (f) inferiorly decrease 1 cm in the shoulder area.

For H&N patients, the body contours were changed in two ways: (a) by ±0.5 cm and ±1 cm in all directions uniformly to simulate weight change in facial area (from the level of pituitary fossa to 1 cm inferior of the most inferior slice of mandible); (b) by ±1 cm and ±2 cm in superior–inferior direction at shoulder position to simulate shoulder positional change, as a result of which the shoulder position in the anterior–posterior direction also changed. These values for prostate^3^, ^4^ and H&N^9^, ^16^ were chosen based on a review of the magnitude of body contour changes. No contour changes were made for OARs and target volumes because the change in the OARs and target volumes was not relevant to the purpose of this study, which was to evaluate the effect of body contour changes on spatial dose distribution. Figure 1 shows examples of body contour deformations for prostate and H&N patients.

**Figure 2:**
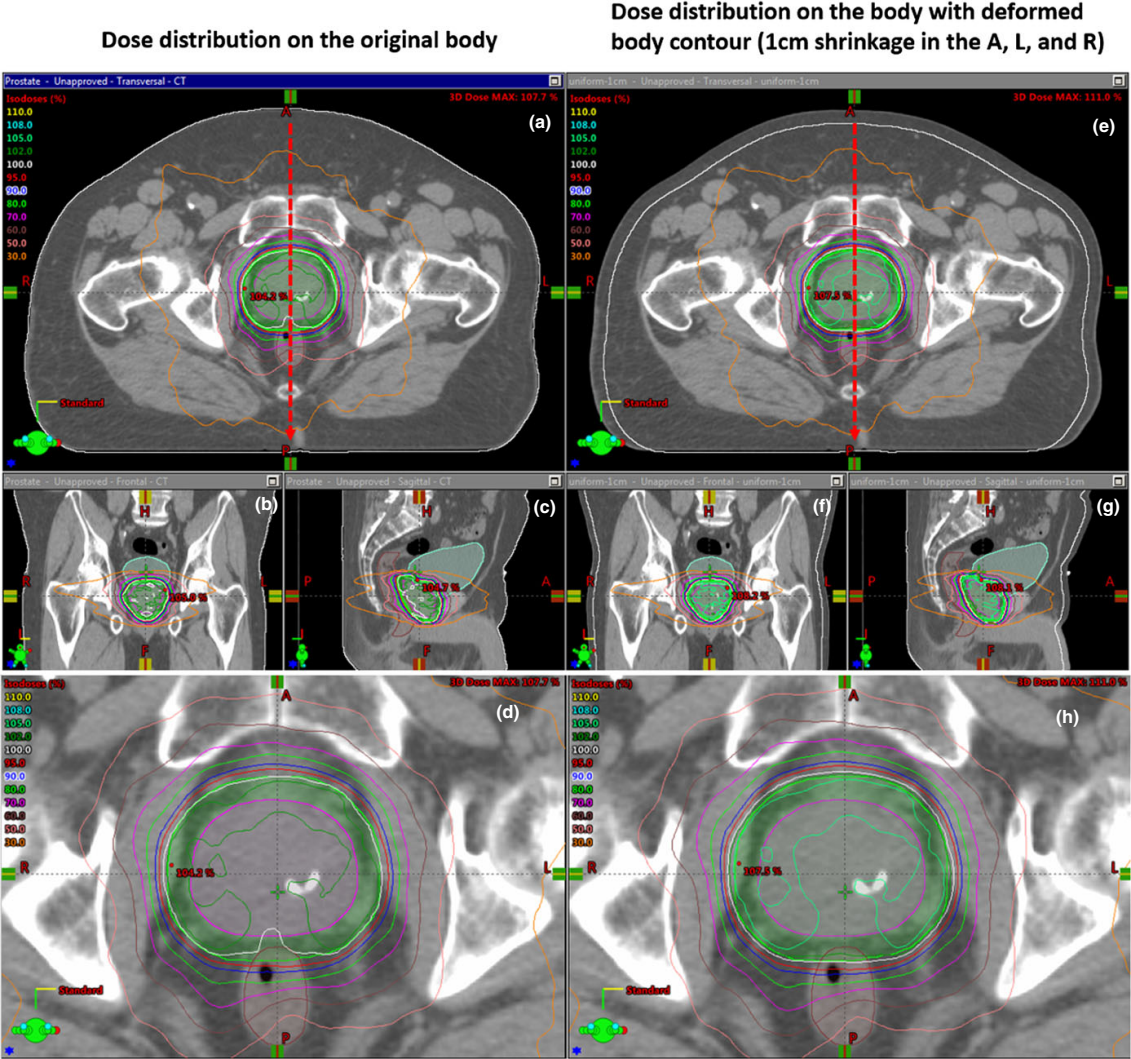
Example dose distributions shown as isodose lines before (a)–(d) and after (e)–(h) external body contour change. The red dashed lines in (a) and (e) represent the direction of the one‐dimensional dose profile chosen. Structures including CTV (magenta), PTV (green), bladder (cyan), and rectum (brown) are contoured in the computed tomography images. (d) and (h) are a magnified view of (a) and (e), respectively.

The original clinical plans were copied to the new CTs and the corresponding doses were calculated (Fig. 2).

### 2.D Dose percentage change and isodose line shift

Body contour changes may lead to the increase or decrease in the dose to the PTV(s) and OARs, which can be quantified by the dose percentage change between the new body and the original body at the same position (*S*):

(1) $$\Delta {\;D}_{S}\left(\%\right)=\left(\frac{D_{new,S}}{D_{\mathrm{original},S}}-1\right)\times 100.$$

The body contour change may also lead to the shrinkage or expansion (shift) of clinically relevant isodose lines (lines connecting the voxels of equal dose), which may be a concern. For example, after body contour change, the 95% isodose line may not fully cover the PTV, leading to increased risk of loco‐regional recurrence, or, a larger portion of the OARs may be covered by the high isodose lines, which may not be acceptable. Therefore, isodose line shift, the distance between the original isodose line and the isodose line on the new body of the same dose level (*D*), was evaluated:

(2) $$\Delta S_{D}\left(\mathrm{mm}\right)=S_{\mathrm{new},D}-S_{\mathrm{original},D}.$$

Both dose percentage change and isodose line shift were acquired from the one‐dimensional dose profiles, considering the balance of computational intensity with clinically relevant outcome. For prostate patients, the dose profiles were chosen in the anterior–posterior direction through the CTV center‐of‐mass (COM) projection at three slices superior to the most inferior slice of the bladder. This slice was chosen because there were visible rectum, bladder, and prostate on this slice and the anterior–posterior direction is considered the most clinically relevant direction, which should reflect dose change to major OARs, namely rectum and bladder. The dose levels studied were 95%, 90%, 80%, 70%, and 60% (percent of prescription dose).

For H&N patients, four dose profiles were chosen: (a) Dose profile measured through high dose CTV COM in anterior, posterior and medial directions to examine dose levels including 95% (min coverage of high dose CTV), 93% (min coverage of high dose PTV), and 78.9% (min coverage of low dose PTV) with the percentages relative to prescription dose; (b) Dose profile connecting high dose CTV COM and spinal cord COM for 48 Gy dose level (max tolerance dose of spinal cord); (c) Dose profile at the most inferior slice of brainstem connecting high dose CTV COM projection and brainstem center on that slice for 54 Gy dose level (max tolerance dose of brainstem). (d) Dose profile in the left–right direction through the low dose CTVs and dose profile connecting spinal cord center to closest low dose CTV at five slices inferior to the most superior slice of shoulders. Those dose profiles were chosen because in those locations the dose distributions were expected to be most sensitive to body contour changes or considered as the worst case scenario. The dose levels in the buildup region (less than 1.5 cm depth) were excluded.

The dose percentage change per centimeter body contour change, Δ*D*(%/*cm*), and isodose line shift per centimeter body contour change, Δ*S*(*mm*/*cm*) were interpolated from dose percentage change and isodose line shift for different depth changes using linear fit.

The rules of thumb were the mean of the patients' medians for different dose levels for the same type of body contour change.

#### 2.D.1 3D gamma index

The gamma index is clinically used for quantitative evaluation of the treatment planning system calculated dose distribution and the measured dose distribution using the acceptability criteria.^17^ In this study, for both prostate and H&N patients, a new structure (PTVandOARs) including all the PTV(s) and OARs was created and the 3D global gamma index was calculated for this structure to quantitatively compare the dose distribution on the original body and the dose distribution on the deformed body for the same plan. For each calculation, the dose distribution on the smaller body contour was the reference to ensure comparison points always existed. Criteria of 3%/3 mm and 5%/3 mm^18^, ^19^ (percentage of maximum reference voxel dose) were used. The final 3D gamma passing rate was the percentage of the examined points in PTV and OARs passing the criteria.^20^ 3D gamma passing rate was computed in PTW VeriSoft^®^ Patient Plan Verification Software (version 6.2). A high gamma passing rate indicated that the dose distributions on the CT images with the original body contour and the deformed body contour were similar; however, physicists and clinicians must also consider that the targets and OARs in the patient may have changed, causing changes in the delivered dose. In this study, 95% was chosen as the pass‐fail threshold for gamma analysis.

## 3 RESULTS

**Figure 3:**
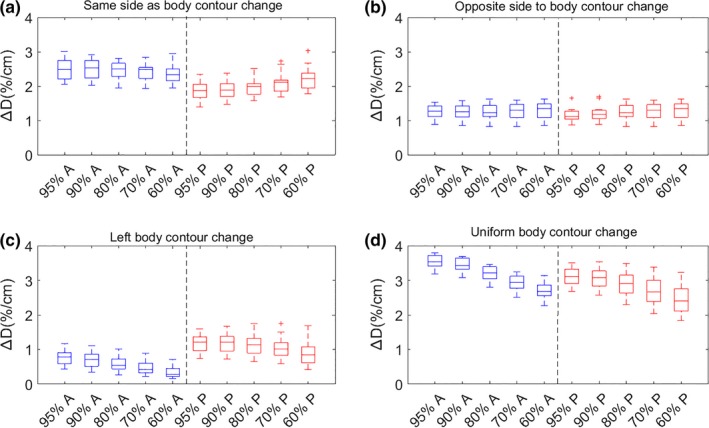
Boxplots of dose percentage change per centimeter body contour change, ∆D (%/cm), for 12 patients' 6 MV volumetric modulated arc therapy prostate plans. Four types of body contour changes and dose levels of 95%, 90%, 80%, 70%, and 60% in the anterior (A, blue boxplots) and posterior (P, red boxplots) directions relative to the prostate center‐of‐mass were examined. [Note: Uniform body contour change in (d) refers to Fig. 1 (d).] (a): ∆D in anterior region due to anterior body contour change and in posterior region due to posterior body contour change; (b): ∆D in anterior region due to posterior body contour change and in posterior region due to anterior body contour change; (c): ∆D in anterior and posterior region due to left body contour change; (d): ∆D in anterior and posterior region due to uniform body contour change. With body contour expansion, ∆D decreases; with body contour shrinkage, ∆D increases.

**Figure 4:**
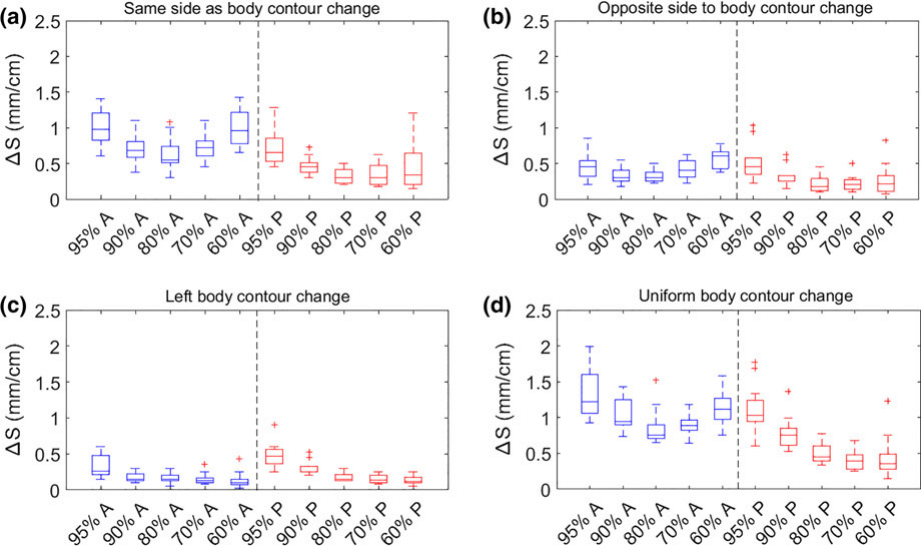
Boxplots of isodose line shift per centimeter body contour change, ∆S (mm/cm), for 12 patients' 6 MV volumetric modulated arc therapy prostate plans, with the same parameters and definitions as Fig. 2. With body contour expansion, the isodose lines get closer to prostate center of mass; with body contour shrinkage, isodose lines get further away from prostate center of mass.

**Figure 5:**
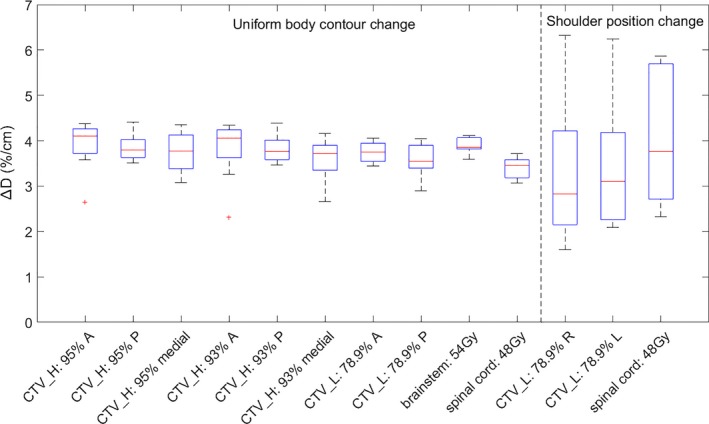
Boxplots of dose percentage change for every centimeter body contour change ∆D (%/cm) for 10 H&N patients' 6 MV volumetric modulated arc therapy plans. Two types of body contour changes (uniform body contour change and shoulder position change) and dose levels of 54, 48 Gy, 95%, 93%, and 78.9% were examined. Generally, With body contour expansion/shoulders moving superiorly, ∆D decreases; with body contour expansion/shoulders moving inferiorly, ∆D increases.

**Figure 6:**
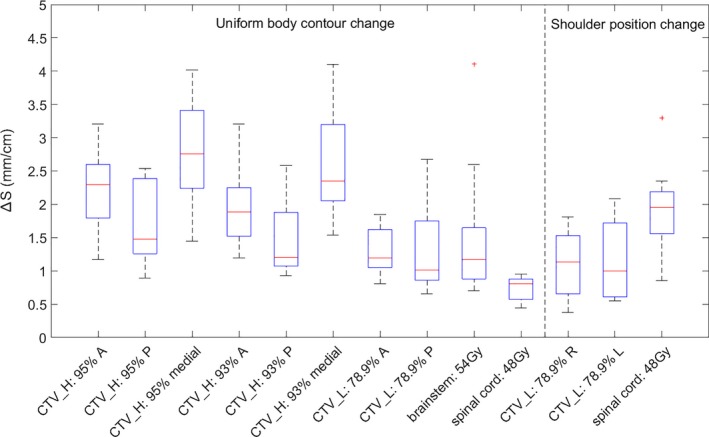
Boxplots of isodose line shift for every centimeter body contour change ∆S (mm/cm) for 10 H&N patients' 6 MV volumetric modulated arc therapy plans. Two types of body contour changes (uniform body contour change and shoulder position change) and dose levels of 54, 48 Gy, 95%, 93%, and 78.9% were examined. Generally, With body contour expansion/shoulders moving superiorly, the isodose lines get closer to the target volumes' center of mass; with body contour shrinkage, isodose lines get further away from target volumes' center of mass.

The results of dose percentage change per centimeter body contour change Δ*D*(%/*cm*) for prostate and H&N plans are shown in Figs. 3 and 5, respectively. The results of isodose line shifts per centimeter body contour change Δ*S*(*mm*/*cm*) for prostate and H&N plans are shown in Figs. 4 and 6, respectively. The rules of thumb are summarized in Table 1. The results of 3D gamma index are shown in Table 2.

**Table 1:** Rules of thumb for dosimetric effect of every centimeter body contour change (results are for 6 MV volumetric modulated arc therapy plans)

Site	Dose percentage change (Δ*D*)	odose line shift (∆S)	Notes
Prostate	3%/cm	0.8 mm/cm	Body contour change in three directions (A, L, and R)
	2%/cm	0.6 mm/cm	Body contour change in one direction (A or P) and dosimetric effect in the same direction as body contour change
	1%/cm	0.3 mm/cm	Body contour change in one direction (A, L, or P) and dosimetric effect in the direction opposite to or orthogonal to body contour change
H&N			
Face	4%/cm	2 mm/cm	Body contour change in four directions (A, P, L, and R)
Shoulder	3%/cm	1 mm/cm	Body contour change in superior–inferior direction

**Table 2:** Mean three‐dimensional (3D) gamma passing rate of the dose distributions on the deformed body and the original body for the same clinical volumetric modulated arc therapy (VMAT) plan with the criteria of 3 mm/3% and 3 mm/5%. The values in the brackets are the standard deviations. Mean 3D gamma passing rate less than 95% is in bold. Results are for 6 MV VMAT plans

Prostate	Anterior body contour change (cm)				Posterior body contour change (cm)			
	−2	−1	1	2	−2	−1	1	2
3 mm/3%	**90.5 (5.0)**	100 (0)	100 (0)	**92.2 (4.7)**	**93.6 (3.9)**	100 (0)	100 (0)	97.7 (2.2)
3 mm/5%	97.9 (1.6)	100 (0)	100 (0)	99.5 (0.5)	99.8 (0.2)	100 (0)	100 (0)	100 (0)

Prostate	Left side body contour change				Uniform body contour change			
3 mm/3%	99.9 (0.2)	100 (0)	100 (0)	100 (0)	**87.7 (6.1)**	**94.5 (1.4)**	95.3 (1.1)	**88.0 (6.1)**
3 mm/5%	100 (0)	100 (0)	100 (0)	100 (0)	**88.1 (5.9)**	100 (0)	100 (0)	**88.6 (5.6)**

H&N	Uniform facial radius change (cm)				Shoulder position change (cm)			
	−1	−0.5	0.5	1	−2	−1	1	2
3 mm/3%	**82.1 (3.8)**	98.0 (3.1)	99.3 (0.4)	**89.4 (2.5)**	**87.0 (4.8)**	95.1 (4.0)	96.6 (2.6)	**88.2 (4.5)**
3 mm/5%	96.0 (3.1)	99.1 (1.2)	99.5 (0.3)	99.1 (0.6)	**92.2 (4.3)**	98.5 (1.5)	98.6 (2.1)	**91.8 (5.0)**

### 3.A Rules of thumb for prostate plans

The medians of Δ*D*(%/*cm*) and Δ*S*(*mm*/*cm*) were consistent between different dose levels and different directions for the same type of body contour change as shown in Figs. 3 and 4, and the variation in the medians were within 1% and 1 mm, meaning it was reasonable to take the average of the median values as the rules of thumb (Table 1). For reference, for a single static 6 MV beam, when the depth changes 1 cm (with the original depth in the range of 10–20 cm for prostate patients' isocentric setup), the dose at the isocenter can change by 4% as derived from TPR ratio and the isodose lines will move about 1 cm (from Varian Golden Beam Data). These values are significantly larger than the rules of thumb in Table 1, which is mainly because in VMAT plans the dose is delivered by 360° arcs with multileaf collimator modulation and the body depth is not changed evenly.

For the prostate data sets in Figs. 3(a) and 4(a), the Δ*D*(%/*cm*) and Δ*S*(*mm*/*cm*) in the anterior region due to anterior body contour change was overall larger than the dose percentage change in the posterior region due to posterior body contour change. This was mainly because a larger portion of the beamlets of the full arcs was experiencing path length change due to anterior body contour change than posterior body contour change [Figs. 1(a) and 1(b)] and the dose deposited in the anterior/posterior region was mostly from beamlets passing through anteriorly/posteriorly. Additionally, a larger portion of the dose was delivered anteriorly than posteriorly because rectum sparing was commonly forced harder than bladder sparing in the optimization process. Comparing Figs. 3(a)–3(c) or 4(a)–4(c), it was obvious that the dosimetric effect in the same direction as body contour change [Figs. 3(a) and 4(a)] was more pronounced than in the opposite direction to body contour change [Fig. 3(b) and 4(b)], both of which were more pronounced than left side body contour change [Fig. 3(c) and 4(c)]. This was due to the fact that the closer a voxel was to a side of the body, the larger portion of the dose would come from the same side of the body and it is more sensitive to body contour change in the same side than the opposite side. This may also be explained by the range of beam path length affected by body contour change and the relative portion of dose delivered in the corresponding direction. Results in Figs. 3(d) or 4(d) can be considered to be a combination of Figs. 3(a) and 3(c) or 4(a) and 4(c) because uniform body contour change represented anterior, left, and right body contour change at the same time.

One trend in Fig. 4 is that high dose isodose lines (e.g., 95% isodose line) and relative low dose isodose lines (e.g., 60% isodose line) shifted more than the intermediate dose isodose lines (e.g., 80% isodose line). This was a result of the shape of the dose profiles. The dose profiles were steepest in the intermediate dose range but flatter in the high dose region and relative low dose region as a result of optimization.

### 3.B Rules of thumb for H&N plans

In Fig. 5, for uniform body contour change, the medians of Δ*D*(%/*cm*) had little variability and the variations of the medians were within 1%. Thus, it was reasonable to take the average of the medians as the rule of thumb (Table 1). This value (4%) was close to the reference value from single beam because the facial geometry was close to a cylinder and the body contour change was happening uniformly. However, the results from patients' shoulder position change had larger variations (as high as 6.5% or as low as 1.5%). This may be due to the fact that the original shoulder position on the p‐CT varied tremendously between patients (the slice examined was five slices inferior to the most superior shoulder contour on the p‐CT) and there may be shoulder contour variability.

In Fig. 6 there were relatively larger variations of Δ*S*(*mm*/*cm*) between H&N patients compared to prostate plans. This may be because the tumor volume and locations for H&N patients were quite different, while the anatomy for prostate patients was relatively similar. While some of the patients had 95% or 93% isodose line shifts less than 1 mm due to 1 cm uniform body contour change, some of the patients had 95% or 93% isodose line shifts more than 4 mm. This may explain the fact that although there was literature claiming the target coverage was affected by anatomical changes (e.g., weight loss),^6^, ^21^ others showed that anatomical changes had minimal effect on target coverage.^7^, ^22^, ^23^

One limitation of the rules of thumb for H&N plans is that these rules cannot assess the dosimetric effect in the buildup region (<1.5 cm from surface), where charged particle equilibrium is absent and the dose fall‐off is very steep. In this region, the dose percentage and isodose line shift are highly dependent on the depth.

### 3.C 3D gamma index

The results for the 3D gamma passing rate are shown in Table 2. For prostate plans, all the dose distributions on the deformed body with 1 cm body contour change at a single side had almost all the examined points agree with the original dose distribution with the criteria of 3 mm/3%; for 2 cm body contour change, more than 95% of the points passed the 3 mm/5% criteria. Additionally, when the body contour was deformed uniformly by 1 cm, the dose distributions passed or were close to pass the 95% threshold with the criteria of 3 mm/3%; while 2 cm uniform body contour deformation could result in about 88% of the examined points passing the 3 mm/5% criteria. Thus, the plans for prostate patients with less than 2 cm single side deformation or less than 1 cm uniform body contour change are unlikely to require further assessment considering the smaller deviation of dose distribution of the target volumes and OARs from the original plans. This agrees with Pair et al.'s conclusion that if there is more than 1 cm source‐surface‐distance deviation, the radiation oncologists need to determine whether to take actions or continue treatment without modifications.^24^

For H&N patients, with 0.5 cm uniform body contour change, more than 95% of the examined points of the dose distributions passed the 3 mm/3% criteria; with 1 cm uniform body contour change, more than 95% of the examined points passed the 3 mm/5% criteria. Therefore, the VMAT plans for oropharyngeal patients who exhibit less than 1 cm uniform body contour change in facial area are unlikely to need further assessment, according to the survey circulated to radiation oncologists in our department about acceptable dose violations.^12^ For shoulder position change, the dose distribution on the original body and the new body was similar if the shoulder only moved 1 cm superiorly or inferiorly while a 2 cm shoulder movement could cause the dose distribution to be significantly different. Thus, VMAT plans for patients with 1 cm or less shoulder positional change may not need to be further assessed. Note that these results were based on the fact that there was no morphological changes within the new body in our study, and for clinical situations with volumetric and positional changes of the PTV and OARs, the dose received by the PTV and OARs should be assessed based on the specific case.

## 4 DISCUSSION

In this study, we find that prostate patients who have body contour changes less than 2 cm at a single side or less than 1 cm uniformly are unlikely to need further assessment. This corresponds to roughly 70% of the treatment fractions of prostate patients according to literature. Stanley et al.^3^ reported that 34% of the 64 analyzed CBCT fractions from five patients showed a body contour change larger than 1 cm in any of the IMRT fields and the majority of the significant changes occurred in the anterior portion of the body contour, while Booth et al.^4^ found that 68% of the 198 analyzed CBCT images from 19 patients were in the range of 0–1 cm, 28% 1–2 cm, and 4% > 2 cm with deviations occurring mostly in the postero‐lateral direction.

For H&N patients, we find that a uniform body contour change less than 1 cm in facial area is unlikely to warrant further assessment due to dose change. However, the anatomical changes may cause the OARs (e.g., parotid glands) to enter high dose regions, which may not be acceptable even with less than 1 cm body contour change. There is evidence showing that weight loss is correlated with body contour changes.^16^, ^25^ Weight loss for H&N patients is well‐known^5^, ^6^, ^7^, ^8^ and body contour change due to weight loss has been quantified in some studies. Yang et al.^21^ reported that the transverse diameter at the odontoid process level decreased on average by 4.6 mm from the first fraction to the 16th fraction and 7.9 mm from the first fraction to the 25th fraction for their 23 patients. Ahn et al.^25^ showed that the average skin separation at the isocenter decreased by 3.2 mm at the 11th fraction, 7.5 mm at the 22nd fraction, and 14 mm at the 33rd fraction for the 23 patients. Tsai et al.^16^ compared the p‐CT with the 2nd CT acquired at the 22nd fraction for 38 H&N patients and they reported the separation distance in each slice of the CT images reduced by an average of 3.2–8.9 mm with the maximum reduction in 9 mm at the level of the 3rd cervical spine.

Moreover, we find that less than a 1 cm shoulder position change in the superior–inferior direction may not warrant plan re‐assessment, depending on institutional tolerance. This means the majority of the H&N patients do not need plan re‐assessment due to shoulder position change if proper shoulder immobilization is used. Neubauer et al.^9^ conducted a study to assess the shoulder position variations for 10 H&N patients using 243 daily CT‐on‐rails scans and 5‐point masks were used for their patient population. They found that the inter‐fractional shoulder variations were on average 2–5 mm in left–right, anterior–posterior and superior–inferior directions, but 2% of the images showed shifts larger than 10 mm (maximum of 20 mm) in anterior–posterior and superior–inferior directions after isocenter correction.

In this study, we evaluated the dosimetric effect of body contour changes and found the rules of thumb for dose percentage change and isodose line shift (Table 1). The dosimetric effect of body contour changes has been discussed in literature. Chow and Liang^26^, ^27^ as well as Pair et al.^24^ studied the dosimetric effect of weight loss (by uniformly shrinking and/or expanding the body contour in anterior, left, and right directions) for prostate IMRT and VMAT plans. Chow and Liang^26^, ^27^ found that the PTV and CTV D99% were increased by 3%–4% and D30% for the rectum and bladder increased by 2%–4% per cm reduced depth. Pair et al.^24^ showed that the target mean dose decreased or increased by 3%–4% per 1‐cm SSD decrease or increase. A similar study for H&N patients was implemented by Chen et al.,^28^ they found that the dose delivered to the target volume significantly increased by 2%–3% for 2–5 mm of body contour shrinkage. There are also other studies reporting the clinically relevant dosimetric parameters for target volume and OARs (parotid gland, spinal cord, and brainstem in particular) based on dose calculations on daily CBCT or CT on‐rail images.^29^ In all of their studies, dosimetric parameters from dose‐volume histograms (DVHs) were acquired while our study emphasizes the impact of body contour change on spatial dose distribution, which is important in plan evaluation or making a clinical decision for dosimetrists, radiation oncologists or medical physicists. On the other hand, our results are consistent with the literature in the sense that the dose percentage change is on the same magnitude (3%–4%) as those dosimetric parameters (e.g., CTV D99%, rectum and bladder D30%) mentioned above.

**Figure 7:**
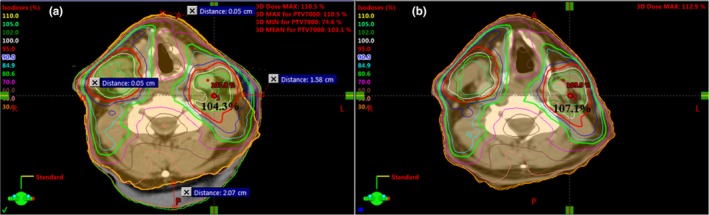
Dose distributions on the planning computed tomography (p‐CT) (a) and the synthetic CT (b, planning CT deformed to the cone‐beam CT on the 28th fraction). The body contour for the 28th fraction is marked on the planning CT (the orange contour) and there is significant body contour change on the left and posterior region.

In this study, the rules of thumbs were derived from “controlled” cases. However, in real clinical situations, the patients' body contour change may not be as regular. For cases like those, we can apply the idea of mean surface distance to approximate the equivalent uniform body contour change and then use the rules of thumb. Figure 7 shows an example for a H&N clinical case and demonstrated how the rules of thumb can be used. From the CBCT taken on the 28th fraction, significant body contour change was noticed with about 1.58 cm shrinkage on the left, negligible change on the right and anteriorly, and 2.07 cm posteriorly. The overall mean body contour shrinkage was about (1.58 + 2.07)/4 = 0.9 cm. According to the rule of thumb, the dose increases by 4% per cm body contour change. The dose at the original point was 104.3%, therefore, the dose at the same point of the new body would be about 104.3% × (0.9 × 4% + 100%) = 108.0% while the actual dose was 107.1%. The two values are close in this example.

VMAT plans are highly conformal and the planning approach can have a significant impact on the plan quality and potentially, the dosimetric response to body contour changes. Thus, we investigated if our rules of thumb can be applied to plans from another cancer center, where different planning approaches are used from our cancer center (e.g., number of arcs, PTV margin, optimization objectives for rectum, etc. but still VMAT in the same treatment planning system). It turned out that the rules of thumbs developed in this manuscript worked well with an accuracy of ±1% for dose percentage change and ±0.5 mm for isodose line shift across 10 randomly selected prostate plans. This is not surprising and the factors we would expect to impact the rules of thumbs are the fraction of dose going through the anatomy (which is influenced by the technique, for example, VMAT vs IMRT, with or without avoidance structure used), and energy.

In the future, daily online re‐planning for prostate and H&N patients in a timely fashion may be achievable. However, currently, as a result of the limited resources in busy cancer centers, it is unlikely that daily online or offline re‐planning will be applied to all patients on a routine basis. The decision on flagging the plans for further assessment and potentially re‐planning is mostly based on the anatomical changes seen on the pre‐treatment images (body contour change as the one that can be easily visualized). Thus, it is still essential to establish ballpark dosimetric consequences that result from body contour changes.

There are some limitations of this study. Firstly, 1D dose profile on a single slice was used for establishing the rules of thumb and a limited number of isodose lines were examined. As a result, the rules of thumb may not work well for low isodose lines (e.g., <50%). Overall, the rules of thumb tend to underestimate the low dose isodose line shift and overestimate the dose percentage change. This is because the dose gradient is shallow in the low dose region. Secondly, the body contour deformation may not accurately model real patient anatomical changes and not all the possible anatomical changes were modelled in this study. For example, the dosimetric effect due to tumor shrinkage for H&N patients was not evaluated because there were only two patients who had bulging tumors. Thus, cautions are needed in applying the rules of thumb under these circumstances. Moreover, although 3 mm/3% and 3 mm/5% are used in this study, which are the commonly used criteria for comparing treatment planning system calculated dose distributions with measured dose distributions, the specific criteria that are relevant for assessing the impact of body contour change may vary depending on the specific clinical scenario in question. To get an accurate estimation, a full dosimetric analysis of body contour changes is necessary. However, the rules of thumb developed in this manuscript can be useful to help the radiation oncologist/physicist/RT staff to make a quick decision on treat or not treat due to body contour changes while the patient is on bed, or to indicate the priority of the full dosimetric calculation, and as a “sanity check” when reviewing such calculations.

## 5 CONCLUSION

In this study, the impact of body contour changes on spatial dose distribution was assessed for prostate and H&N VMAT plans. Rules of thumb for dose percentage change and isodose line shift due to different body contour changes were provided for non‐buildup regions. In addition, guidelines were given for patients who underwent body contour changes but were unlikely to require plan re‐assessment. However, the judgment is dependent on center‐specific tolerances for dose deviations.

## CONFLICT OF INTEREST

The authors declare that they have no conflict of interest.
